# Appointment

**Published:** 1856-09

**Authors:** 


*Appointment.*—Dr. T. G. Richardson, late Demonstrator of Anatomy in the University of Louisville, has been appointed Professor of Anatomy in the Pennsylvania Medical College, Philadelphia, *vice* Prof. J. M. Allen, resigned. The new professor is the author of Richardson's Anatomy, published a few years since and highly praised at that time. He has been for a long time engaged as a teacher of practical anatomy, and brings to his new station a high order of qualification, and, as we learn from others who know him well, those qualities of a high-toned gentleman which will render him a desirable colleague to his new associates.

